# Growth-Inhibitory Activity of Raw and Pasteurized Donkey Milk Against Clinically Relevant Gram-Negative Isolates with Different Antimicrobial Resistance Profiles

**DOI:** 10.3390/ani16131996

**Published:** 2026-06-29

**Authors:** Anika Trudić, Ljubiša Šarić, Dragana Plavšić, Olja Todorić, Miloš Pelić, Ivana Čabarkapa, Dragana Tomanić

**Affiliations:** 1 Faculty of Medicine, University of Novi Sad, Hajduk Veljkova 3, 21000 Novi Sad, Serbia; anika.trudic@mf.uns.ac.rs; 2 Institute for Pulmonary Diseases of Vojvodina, Put Doktora Goldmana 4, 21204 Sremska Kamenica, Serbia; 3 Institute of Food Technology in Novi Sad, University of Novi Sad, Bulevar Cara Lazara 1, 21000 Novi Sad, Serbia; ljubisa.saric@fins.uns.ac.rs (L.Š.); dragana.plavsic@fins.uns.ac.rs (D.P.); olja.todoric@fins.uns.ac.rs (O.T.); milos.pelic@fins.uns.ac.rs (M.P.); ivana.cabarkapa@fins.uns.ac.rs (I.Č.)

**Keywords:** donkey milk, antimicrobial activity, antimicrobial resistance, Enterobacterales

## Abstract

Antimicrobial resistance (AMR) is a major global health challenge affecting human and animal health, food safety, and the effectiveness of antimicrobial therapy. Therefore, this study evaluated the antimicrobial activity of raw (RDM) and pasteurized donkey milk (PDM) against clinically relevant Gram-negative bacteria with different AMR profiles. Antimicrobial activity against ten clinical Enterobacterales isolates, including CTX-M-, OXA-48-, KPC-, NDM-, and VIM-producing strains, was assessed by monitoring bacterial growth kinetics in artificially contaminated milk samples during 8 h of incubation at 37 °C. Pasteurized cow milk (CM) and nutrient broth (NB) served as controls. The strongest antimicrobial effects were observed against OXA-48-producing *Klebsiella pneumoniae* A4 and CTX-M-producing *Escherichia coli* A1, whereas *Citrobacter koseri* F5 showed the lowest susceptibility. After 8 h, *K. pneumoniae* A4 counts remained below 2 log CFU/mL in RDM compared with >6 log CFU/mL in NB. Significant effects of milk type, incubation time, and their interaction on bacterial growth were confirmed (*p* < 0.001). Donkey milk significantly inhibited bacterial growth compared with NB and CM, while some differences between RDM and PDM were observed depending on the bacterial isolate. These findings support donkey milk as a bioactive matrix with potential antimicrobial applications.

## Simple Summary

Antimicrobial resistance is one of the greatest global health challenges because many bacteria are becoming increasingly difficult to treat with conventional antimicrobial agents. Gram-negative bacteria such as *Escherichia coli* and *Klebsiella pneumoniae* are especially important because they can cause severe infections and often carry resistance mechanisms that reduce the effectiveness of available therapies. Therefore, there is growing interest in natural products with potential antimicrobial activity. Donkey milk has attracted scientific attention due to its rich content of natural bioactive compounds, including lysozyme, lactoferrin, and antimicrobial peptides. In this study, we investigated whether raw and pasteurized donkey milk could slow the growth of clinically important Gram-negative bacteria with different antimicrobial resistance profiles. The results showed that donkey milk delayed bacterial growth compared with nutrient broth and cow milk. Some differences in antimicrobial activity were observed between raw and pasteurized donkey milk depending on the bacterial isolate tested. These findings indicate that donkey milk could represent a valuable natural bioactive food with potential applications in food microbiology, functional nutrition, and complementary antimicrobial research, as well as in the development of novel antimicrobial strategies.

## 1. Introduction

Antimicrobial resistance (AMR) represents one of the most serious global public health threats, substantially reducing the effectiveness of currently available antimicrobial therapies and contributing to increased morbidity, mortality, and healthcare-associated costs worldwide [1]. In 2021, bacterial AMR was estimated to be associated with approximately 4.71 million deaths worldwide, including 1.14 million deaths directly attributable to AMR [2]. Due to its growing clinical and socioeconomic impact, the World Health Organization (WHO) has identified AMR among the most serious threats to global health [3]. Particularly concerning is the rapid emergence and dissemination of multidrug-resistant (MDR) Gram-negative bacteria, especially Enterobacterales producing extended-spectrum β-lactamases (ESBLs), including those carrying CTX-M enzymes, and carbapenemases, which are associated with limited therapeutic options and poor clinical outcomes [4]. These isolates are frequently MDR, as CTX-M and carbapenemase genes are often carried on mobile genetic elements together with additional resistance determinants affecting other antimicrobial classes. Carbapenem-resistant and third-generation cephalosporin-resistant Enterobacterales have therefore been classified by the WHO among the highest-priority bacterial pathogens requiring the development of new antimicrobial strategies [5]. Clinically important species such as *Escherichia coli*, *Klebsiella pneumoniae*, *Proteus mirabilis*, *Serratia marcescens*, and related Enterobacterales are frequently associated with urinary tract infections, pneumonia, bloodstream infections, and other healthcare-associated infections [6]. Consequently, increasing scientific attention has been directed toward naturally derived bioactive systems with the potential to limit bacterial growth.

Among natural bioactive products, milk from non-bovine species has attracted increasing scientific attention due to its unique nutritional composition and biologically active compounds. Donkey milk, in particular, has increasingly been recognized as a functional food with biological properties extending beyond its basic nutritional value [7,8]. Its unique biochemical composition is characterized by high concentrations of lysozyme, lactoferrin, immunoglobulins, bioactive peptides, and unsaturated fatty acids, which may contribute to its antimicrobial, antioxidant, anti-inflammatory, and immunomodulatory activities [9,10]. Previous studies have demonstrated the inhibitory activity of donkey milk against various bacterial species, suggesting its potential application as a functional food and natural antimicrobial source. Zhang et al. [11] reported that donkey milk exerted bactericidal activity against *Shigella dysenteriae*, reducing viable counts below the detection limit, while *Salmonella choleraesuis* populations remained approximately 3 log CFU/mL lower than those observed in nutrient broth after 48 h of incubation. Furthermore, Šarić et al. [12] demonstrated a strong inhibitory effect of donkey milk against a clinical isolate of *K. pneumoniae*, with bacterial counts decreasing by approximately 2 log CFU/mL in samples with higher lactoferrin concentrations. Additional studies have also reported inhibitory activity against *Listeria monocytogenes* and *Staphylococcus aureus* [13]. The antimicrobial properties of donkey milk are primarily associated with the synergistic action of several bioactive compounds [7]. Lysozyme is considered one of the key antimicrobial components due to its ability to hydrolyze peptidoglycan in bacterial cell walls, while lactoferrin exerts bacteriostatic activity through iron sequestration and direct membrane interactions. In addition, naturally occurring antimicrobial peptides and fatty acids may further contribute to bacterial growth inhibition [14].

Despite the growing interest in donkey milk as a biologically active dairy matrix, available studies have predominantly focused on standard laboratory strains or a limited number of foodborne bacteria. Data regarding its activity against clinically relevant Gram-negative isolates, particularly those exhibiting AMR phenotypes, remain scarce. Additional investigations using clinically derived isolates are therefore needed to better characterize the growth-inhibitory potential of donkey milk against bacteria of medical relevance. Accordingly, the aim of this study was to evaluate the in vitro antimicrobial activity of raw (RDM) and pasteurized donkey milk (PDM) against clinically relevant Gram-negative bacteria with different AMR profiles. An additional objective was to provide preliminary insight into whether pasteurization influences the growth-inhibitory activity of donkey milk.

## 2. Materials and Methods

### 2.1. Bacteria Isolation and Identification

Clinical Gram-negative isolates used in this study were obtained from patients treated at the Institute for Pulmonary Diseases of Vojvodina, Serbia, a tertiary care hospital, and were processed in the Department for Microbiological Diagnostics as part of routine diagnostic procedures. The isolates originated from different clinical specimens, including wound swabs, sputum, tracheal aspirate, urine, and blood cultures.

The bacterial isolates were selected to represent clinically relevant Enterobacterales with diverse AMR profiles. MDR isolates were purposefully selected to include highly resistant representatives of different species and clinically important β-lactamase mechanisms, including CTX-M, OXA-48, KPC, NDM, and VIM. Susceptible or non-ESBL/non-carbapenemase-producing clinical isolates were included as comparators and were randomly selected from isolates recovered from infections in hospitalized patients. The analyzed isolates included *E. coli* A1, C8, and F8, *Klebsiella oxytoca* A3, *K. pneumoniae* A4 and C5, *S. marcescens* A6, *Providencia stuartii* F4, *Citrobacter koseri* F5, and *P. mirabilis* F9 (Table 1).

Primary cultivation was performed on standard culture media selected according to the type of clinical specimen and routine laboratory protocols. Bacterial identification was performed by matrix-assisted laser desorption/ionization time-of-flight mass spectrometry using the VITEK MS system, according to the manufacturer’s instructions (bioMérieux, Marcy-l’Étoile, France). Before experimental testing, all isolates were stored in duplicate at −80 °C and were recovered by subculturing on appropriate non-selective media.

### 2.2. Antimicrobial Susceptibility Testing of Isolates

Antimicrobial susceptibility testing was performed using the VITEK 2 Compact automated system, according to the manufacturer’s instructions (bioMérieux, Marcy-l’Étoile, France). The tested antimicrobial agents included β-lactams, β-lactam/β-lactamase inhibitor combinations, cephalosporins, carbapenems, aminoglycosides, fluoroquinolones, trimethoprim/sulfamethoxazole, ceftazidime/avibactam, and colistin, depending on the species and the routinely applied antimicrobial susceptibility testing panel. Results were interpreted according to the current EUCAST clinical breakpoints valid at the time of testing [15].

The presence of extended-spectrum β-lactamase and carbapenemase production was further investigated using lateral flow immunochromatographic assays. CTX-M production was assessed using the RESIST CTX-M lateral flow assay, while carbapenemase production was detected using the OKNVI RESIST-5 assay, both according to the manufacturer’s instructions (Coris BioConcept, Gembloux, Belgium). The included isolates represented different AMR profiles, comprising a CTX-M-producing *E. coli* isolate, OXA-48-producing *K. pneumoniae* and *P. stuartii* isolates, a KPC-producing *K. pneumoniae* isolate, NDM-producing *C. koseri* and *E. coli* isolates, and a VIM-producing *P. mirabilis* isolate. Several isolates without detected CTX-M or carbapenemase production were also included as comparators.

### 2.3. Milk Sampling Procedure

#### 2.3.1. Sample Collection

Bulk milk samples were collected from the Special Nature Reserve Zasavica, Serbia. The study included 15 clinically healthy Domestic Balkan donkeys aged 4–10 years, managed under conditions consistent with established good dairy husbandry and animal welfare guidelines (AWIN/FAO) [16,17]. Animals were housed in group systems with access to shelter, clean water ad libitum, and a forage-based diet and were regularly monitored for health status [16]. Animals were in different lactation stages, ranging from 75 to 210 days postpartum. Before the morning milking, udders were washed with cold running water and dried using clean towels, while the first milk streams were discarded to minimize potential contamination. Milk was collected aseptically into sterile 0.5 L plastic bottles and immediately frozen at −18 °C in standard household freezers, reflecting routine farm management practice.

A single bulk milk batch was obtained by pooling milk collected from all 15 donkeys included in the study. Because several consecutive days were required to obtain a sufficient volume of milk for all planned experiments, milk from successive milkings was collected and frozen. After the required volume had been obtained, the frozen milk samples were thawed under refrigeration conditions (4 °C), pooled to obtain a homogeneous bulk milk batch, and stored at 4 °C until experimental use. Consequently, both raw donkey milk (RDM) and pasteurized donkey milk (PDM) used in the antimicrobial assays originated from the same pooled bulk milk batch.

#### 2.3.2. Milk Pasteurization

Frozen raw bulk milk samples were transported under refrigerated conditions to the laboratory. Prior to thermal treatment, the milk was allowed to thaw at room temperature. Pasteurization was performed using the low-temperature long-time (LTLT) method at 63 °C for 30 min, with milk processed in sterile test tubes in a water bath (Raypa, Terrassa, Barcelona, Spain).

### 2.4. Antimicrobial Activity Assay

The antimicrobial activity assay was conducted using RDM and PDM samples artificially inoculated with the following clinical isolates obtained from the Institute for Pulmonary Diseases of Vojvodina: *K. pneumoniae* A4 and C5, *K. oxytoca* A3, *E. coli* C8, F8, and A1, *S. marcescens* A6, *C. koseri* F5, *P. mirabilis* F9, and *P. stuartii* F4. Following overnight cultivation on blood agar (Himedia, Mumbai, India) at 37 ± 1 °C, material from well-isolated colonies of each strain was suspended in 0.1% peptone saline and homogenized by vortexing. The turbidity of each bacterial suspension was adjusted to 0.5 McFarland standard using a DEN-1 densitometer (Biosan, Riga, Latvia), after which serial decimal dilutions were prepared in 0.1% peptone saline for further analysis.

RDM and PDM samples were artificially contaminated at a contamination level of 10^2^ CFU/mL using appropriate dilutions of the bacterial suspensions. Contaminated samples were incubated at 37 °C for 8 h. Changes in the number of tested bacteria were monitored every 1 h by the pour plate method using Violet Red Bile Glucose agar (VRBG) (Himedia, Mumbai, India). Inoculated plates were incubated at 37 ± 1 °C for 24 h, after which the number of colonies was determined. Nutrient broth (NB) (Himedia, Mumbai, India) and pasteurized cow milk (CM) inoculated at the same level of contamination (10^2^ CFU/mL) were used as positive controls, while non-inoculated RDM and PDM served as negative controls. Pasteurized CM used as a control was a commercially available milk (Moja Kravica, Imlek, Belgrade, Serbia) containing 2.8% milk fat. Three independent antimicrobial assays were performed for each selected bacterial strain.

### 2.5. Statistical Analysis

Descriptive statistics were calculated for all measured variables and are presented as mean values and standard deviations (mean ± SD). For each bacterial isolate separately, the effects of milk type and incubation time on the log-transformed bacterial count were evaluated using a two-way analysis of variance (ANOVA), with milk type and incubation time included as fixed factors. In addition to the main effects, the interaction between milk type and incubation time was tested in order to determine whether temporal changes in bacterial count differed among the analyzed milk matrices. Before performing ANOVA, the assumptions of normality and homogeneity of variances were assessed using the Shapiro–Wilk test and Levene’s test. When significant effects were detected, pairwise comparisons between factor levels were performed using Tukey’s honestly significant difference (HSD) post hoc test. Effect sizes were expressed using omega-squared (ω^2^) coefficients. The significance threshold was set at *p* < 0.05. All statistical analyses were carried out in R 4.6.0.

## 3. Results

### 3.1. Antimicrobial Resistance Profiles of Clinical Isolates

The analyzed Gram-negative isolates showed considerable variability in antimicrobial susceptibility profiles (Table 2). The CTX-M-producing *E. coli* isolate was resistant to third- and fourth-generation cephalosporins, fluoroquinolones, gentamicin, tobramycin, and trimethoprim/sulfamethoxazole, while remaining susceptible to carbapenems, amikacin, and ceftazidime/avibactam. OXA-48- and KPC-producing *K. pneumoniae* isolates exhibited resistance to most β-lactams, carbapenems, fluoroquinolones, and several aminoglycosides. The NDM-producing *C. koseri* and *E. coli* isolates, as well as the VIM-producing *P. mirabilis* isolate, demonstrated the highest resistance levels, including resistance to carbapenems and multiple additional antimicrobial classes. Overall, CTX-M- and carbapenemase-producing isolates displayed MDR phenotypes, reflecting resistance to β-lactams together with resistance to one or more additional antimicrobial classes. In contrast, *K. oxytoca*, *S. marcescens*, and one non-resistant *E. coli* isolate remained susceptible to most tested antimicrobial agents.

### 3.2. Growth Kinetics in Different Milk Matrices

**Figure 1:**
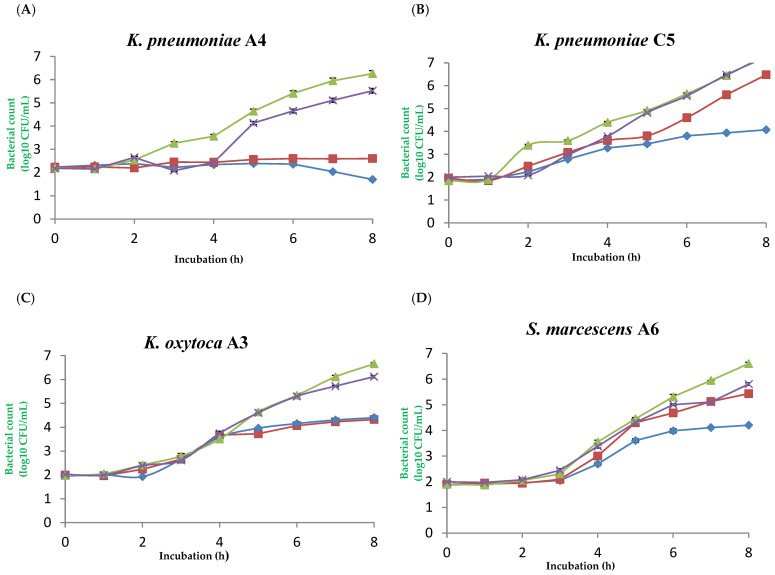

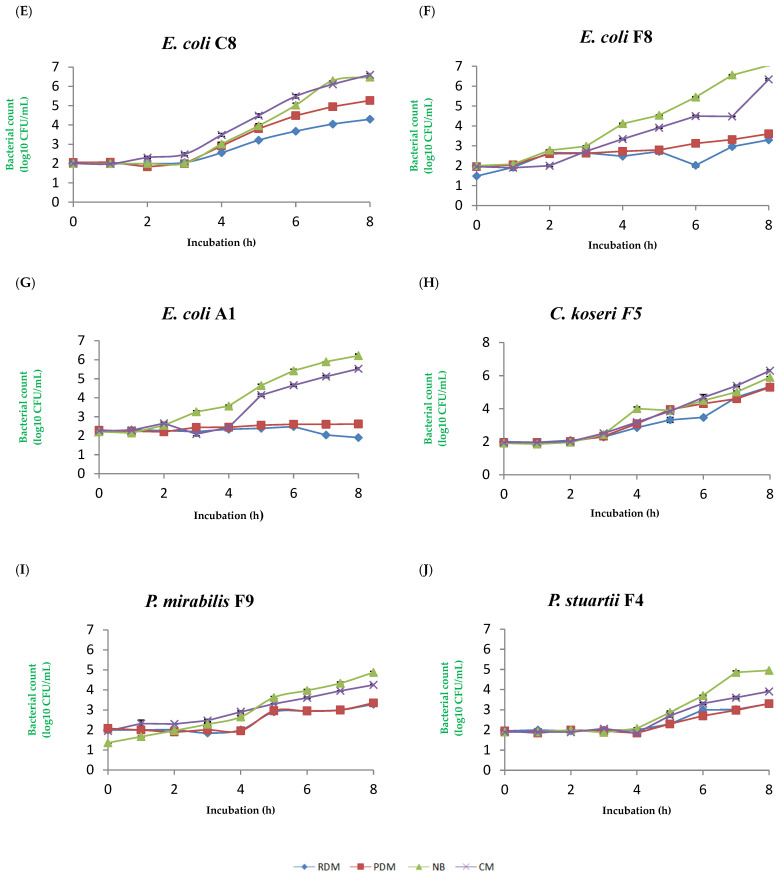
Growth kinetics of Gram-negative isolates during incubation in different matrices. (**A**) *Klebsiella pneumoniae* A4; (**B**) *Klebsiella pneumoniae* C5; (**C**) *Klebsiella oxytoca* A3; (**D**) *Serratia marcescens* A6; (**E**) *Escherichia coli* C8; (**F**) *Escherichia coli* F8; (**G**) *Escherichia coli* A1; (**H**) *Citrobacter koseri* F5; (**I**) *Proteus mirabilis* F9; (**J**) *Providencia stuartii* F4. RDM—raw donkey milk; PDM—pasteurized donkey milk; NB—nutrient broth; CM—cow milk. Data points represent mean values, while error bars indicate standard deviation (SD).

Bacterial growth dynamics differed among the analyzed Gram-negative isolates and depended on both incubation time and matrix type (Figure 1). The strongest growth-inhibitory effects of donkey milk were observed for the OXA-48-producing *K. pneumoniae* A4 and the CTX-M-producing *E. coli* A1 isolates, which maintained substantially lower bacterial counts throughout incubation in both RDM and PDM compared with NB and CM. In particular, after 8 h of incubation, *K. pneumoniae* A4 counts remained below 2 log CFU/mL in RDM and around 2.6 log CFU/mL in PDM, whereas bacterial proliferation in NB exceeded 6 log CFU/mL. A similar growth pattern was observed for *E. coli* A1, where bacterial counts in donkey milk matrices remained markedly lower than in NB and CM throughout incubation. A transient decrease in bacterial count was observed for *E. coli* F8 at the 6 h sampling point in one matrix; however, this effect was not sustained during subsequent incubation and was followed by continued bacterial proliferation, suggesting a temporary fluctuation rather than a stable inhibitory response. Moderate reductions in bacterial proliferation were also observed for *P. mirabilis* F9 and *P. stuartii* F4, indicating partial growth-inhibitory activity of donkey milk against these isolates.

In contrast, certain carbapenemase-producing isolates, particularly the NDM-producing *C. koseri* F5 and *E. coli* F8, as well as the KPC-producing *K. pneumoniae* C5, demonstrated progressive growth regardless of matrix type, although bacterial counts generally remained lower in donkey milk matrices than in NB during prolonged incubation. Similarly, *K. oxytoca* A3 and *S. marcescens* A6 exhibited gradual bacterial proliferation in all analyzed matrices, with higher final bacterial counts observed in NB and CM compared with RDM and PDM. No bacterial growth was observed on VRBG agar inoculated with the negative controls. Overall, the obtained findings indicate isolate-dependent antimicrobial activity of donkey milk and suggest that milk matrix composition significantly influenced bacterial growth dynamics during incubation. Detailed descriptive statistics (mean ± SD) for all isolates and incubation conditions are presented in Supplementary Table S1.

### 3.3. Effects of Milk Matrix and Incubation Time

Following the descriptive analysis, ANOVA was performed for each analyzed isolate in order to evaluate the effects of milk type, incubation period, and their interaction on log-transformed bacterial counts (Table 3). ANOVA demonstrated that milk type, incubation period, and their interaction significantly affected log-transformed bacterial counts for all analyzed isolates (all *p* < 0.001). The dominant source of variability was determined based on ω^2^ values, reflecting effect size rather than F-values alone. For most isolates, incubation period represented the dominant source of variability, as indicated by the highest ω^2^ coefficients. In contrast, for *E. coli* A1, the ω^2^ values for milk type, incubation period, and their interaction were highly similar, indicating comparable contributions of all three factors. A similar pattern was observed for *K. pneumoniae* A4, where the interaction term showed the highest ω^2^ value, although its contribution remained comparable to those of both main effects. These findings indicate that bacterial counts were influenced not only by incubation time but also by milk type, and that the pattern of change over time differed among the tested milk matrices.

### 3.4. Comparative Growth-Inhibitory Activity of Milk Matrices

Post hoc Tukey HSD analysis confirmed significant differences between most milk matrices and incubation intervals across the analyzed isolates. In general, NB and CM supported significantly higher bacterial growth, whereas RDM and PDM were associated with lower bacterial counts and slower proliferation. All pairwise matrix comparisons were significant for *K. pneumoniae* A4, *E. coli* C8, *E. coli* A1, *S. marcescens* A6, *C. koseri* F5, and *P. mirabilis* F9 (all *p* < 0.001). In contrast, no significant differences between RDM and PDM were observed for *K. oxytoca* A3 and *P. stuartii* F4. For most isolates, bacterial counts remained relatively stable during the earliest incubation intervals, followed by significant increases during prolonged incubation, particularly in NB and CM matrices.

## 4. Discussion

The present study demonstrated that both RDM and PDM were associated with slower bacterial growth compared with NB and CM, although the magnitude of this effect depended on the bacterial isolate and its AMR profile. The most pronounced growth-inhibitory effect was observed for the OXA-48-producing *K. pneumoniae* A4 and the CTX-M-producing *E. coli* A1, whereas the NDM-producing *C. koseri* F5 and *E. coli* F8, together with the KPC-producing *K. pneumoniae* C5, exhibited progressive growth in all tested matrices despite lower bacterial counts in donkey milk compared with NB and CM.

These findings support the growing body of evidence suggesting that donkey milk represents not only a nutritionally valuable food but also a source of biologically active compounds with potential antimicrobial effects [18,19,20,21]. The lower bacterial counts observed in donkey milk matrices compared with CM indicate that donkey milk provided less favourable conditions for bacterial growth, likely due to the presence of naturally occurring antimicrobial compounds such as lysozyme and lactoferrin. The observed growth-inhibitory effects are probably associated with the complex bioactive composition of donkey milk [22]. Previous studies have suggested that the antimicrobial activity of donkey milk results from the combined action of multiple bioactive compounds, including lysozyme, lactoferrin, antimicrobial peptides, and fatty acids, which may collectively contribute to the reduced bacterial growth observed in several clinically relevant isolates [13,23]. Mechanistically, the antimicrobial activity of donkey milk is believed to result from the synergistic action of several bioactive components. Lysozyme hydrolyzes β-(1,4)-glycosidic bonds within bacterial peptidoglycan, compromising cell wall integrity, while lactoferrin exerts antimicrobial effects through iron sequestration and direct interactions with bacterial membranes [7,23,24,25]. In addition, naturally occurring antimicrobial peptides derived from milk proteins may disrupt membrane structure and interfere with essential cellular processes [23,26]. Unsaturated fatty acids present in donkey milk, including oleic and linoleic acids, have also been reported to affect bacterial membrane integrity and metabolism, thereby contributing to the overall antimicrobial activity of the milk matrix [13].

The antimicrobial effects observed in this study are in agreement with previous reports describing donkey milk as a biologically active matrix capable of limiting bacterial proliferation. Šarić et al. [12] demonstrated inhibitory activity of donkey milk against a clinical isolate of *K. pneumoniae*, particularly in samples containing higher lactoferrin concentrations. In our study, a comparable pattern was observed for the OXA-48-producing *K. pneumoniae* A4 isolate, which maintained markedly lower bacterial counts in both RDM and PDM compared with NB and CM. Šarić et al. [27] reported that the reference foodborne bacterial strain *E. coli* was among the most susceptible Gram-negative bacteria to the natural antimicrobial compounds present in donkey milk, whereas Abd El-Hack et al. [28] demonstrated that, although donkey milk exerted measurable antimicrobial activity against *E. coli*, complete bacterial inhibition was not consistently achieved during prolonged incubation. Similarly, the analyzed CTX-M-producing *E. coli* A1 isolate exhibited markedly reduced growth dynamics in both RDM and PDM compared with NB and CM, despite progressive bacterial proliferation during prolonged incubation.

Interestingly, resistance to conventional antimicrobial agents did not appear to directly predict the growth response in donkey milk matrices. This is relevant because CTX-M- and carbapenemase-producing Enterobacterales are commonly MDR, reflecting the frequent co-occurrence of β-lactamase genes with resistance determinants affecting other antimicrobial classes. Although several carbapenemase-producing isolates, including the KPC-producing *K. pneumoniae* C5 and the NDM-producing *C. koseri* F5 and *E. coli* F8, exhibited progressive growth during incubation, lower bacterial counts were still generally observed in RDM and PDM compared with NB and CM. The transient decrease observed for *E. coli* F8 at 6 h should be interpreted with caution. Considering the low initial inoculum and the inherent variability of plate-count enumeration methods, this isolated reduction most likely reflects normal experimental variation rather than a biologically meaningful inhibitory event. This interpretation is supported by the subsequent recovery and continued growth of the isolate during later incubation intervals. Moreover, the OXA-48-producing *K. pneumoniae* A4 and CTX-M-producing *E. coli* A1 demonstrated considerably reduced proliferation in donkey milk matrices despite their clinically relevant resistance phenotypes. These observations suggest that the antimicrobial mechanisms associated with donkey milk differ from those targeted by conventional antimicrobial agents and may therefore remain active independently of the bacterial AMR phenotype [24]. This observation is supported by previous studies demonstrating that milk-derived bioactive compounds such as lactoferrin exert antimicrobial activity through mechanisms different from those targeted by conventional antimicrobial agents, including iron sequestration, membrane destabilization, and interference with bacterial virulence factors [25,26]. However, because each resistance mechanism was represented by only a small number of isolates, these findings should not be interpreted as evidence of a direct association between a specific β-lactamase type and MDR profile and susceptibility to donkey milk. Rather, they indicate that the growth response was isolate-dependent and may be influenced by bacterial species, strain-specific characteristics, and milk composition.

Nevertheless, all analyzed isolates demonstrated some degree of bacterial growth during incubation, indicating that donkey milk did not completely inhibit bacterial proliferation. This finding is not unexpected considering the intrinsic structural features of Gram-negative bacteria, particularly the presence of an outer membrane in the cell wall that acts as an effective permeability barrier and limits penetration of antimicrobial compounds [29]. Previous studies demonstrated variable antimicrobial activity of donkey milk against different bacterial species and strains, with susceptibility patterns depending on both bacterial characteristics and milk composition [11,27,30,31,32]. Therefore, the obtained data should primarily be interpreted as evidence of bacterial growth inhibition rather than complete bacterial inhibition.

A direct comparison between RDM and PDM indicated that the effect of pasteurization was isolate-dependent. Significant differences between RDM and PDM were observed for several tested isolates, with RDM generally showing stronger growth-inhibitory activity, particularly against *K. pneumoniae* A4 and *E. coli* A1. In contrast, no significant differences were detected for *K. oxytoca* A3 and *P. stuartii* F4, suggesting that the antimicrobial activity of donkey milk was largely preserved after pasteurization for these isolates. An additional important observation of this study was the relatively similar behavior of RDM and PDM for several analyzed isolates. Although certain differences between RDM and PDM were observed, pasteurization did not completely eliminate the antimicrobial potential of donkey milk. This observation is consistent with previous reports indicating that donkey milk retains substantial lysozyme activity after thermal treatment due to the relatively high thermal stability of this enzyme [33]. Cosentino et al. [34] further demonstrated that standard pasteurization conditions preserve a considerable proportion of lysozyme antimicrobial activity in donkey milk. The observed preservation of antimicrobial activity following pasteurization should be considered in relation to the thermal treatment applied. The present study evaluated antimicrobial activity after LTLT pasteurization, whereas commercial milk processing more commonly relies on high-temperature short-time (HTST) treatment or ultra-high-temperature (UHT) treatments. Previous studies have shown that lysozyme, one of the major antimicrobial proteins in donkey milk, is relatively heat stable and can retain substantial activity after conventional pasteurization treatments [18,35]. However, more intensive heat processing may affect the activity of lysozyme as well as other bioactive compounds, including lactoferrin and certain antimicrobial peptides. Therefore, the antimicrobial effects observed in the present study may not be directly transferable to HTST-processed donkey milk or UHT-processed donkey milk, and future studies should compare different pasteurization regimes to determine their impact on antimicrobial activity and bioactive component preservation.

Importantly, the biological relevance of donkey milk-associated bioactivity is additionally supported by a prospective clinical pilot study by Kolarov et al. [36] involving patients with community-acquired pneumonia, in which supplementation with pasteurized donkey milk was associated with faster reductions in inflammatory biomarkers, improved radiological recovery, and shorter hospitalization duration. Although these clinical observations cannot be attributed solely to direct antimicrobial effects, the in vitro observations further support the potential role of donkey milk as a complementary bioactive dairy matrix.

Although the obtained results demonstrated measurable growth-inhibitory effects of donkey milk against several clinically important isolates, certain limitations should be acknowledged. The number of analyzed isolates was limited, and the study did not determine the minimum inhibitory concentrations or identify the specific bioactive compounds responsible for the observed antimicrobial effects. In addition, the chemical composition of the donkey milk was not characterized, and the concentrations of lysozyme, lactoferrin, and other bioactive components were not quantified. Consequently, the relationship between milk composition and antimicrobial activity could not be assessed. Furthermore, physicochemical parameters such as pH were not systematically correlated with bacterial growth dynamics.

An additional limitation is that the study was performed using a single bulk milk batch obtained from 15 donkeys; therefore, individual animal variability and potential differences related to lactation stage could not be evaluated. It should also be noted that the observed differences between donkey milk and cow milk cannot be attributed exclusively to antimicrobial compounds, as the two matrices differ in several physicochemical and nutritional characteristics, including protein, fat, lactose, and mineral content, all of which may influence bacterial growth. Therefore, the specific contribution of individual milk components to the observed antimicrobial effects remains unclear.

Another limitation is that the potential effects of freezing and thawing on the indigenous milk microflora and bioactive compounds were not evaluated. Although lysozyme in donkey milk is considered relatively stable during processing [34,35], the impact of freezing and thawing on the overall bioactive profile and its contribution to antimicrobial activity remains unknown. Finally, the experiments were conducted under in vitro conditions, which may not fully reflect the complexity of in vivo microbial interactions. Future studies should therefore focus on characterizing the individual antimicrobial components of donkey milk, evaluating their synergistic effects, and clarifying the mechanisms underlying bacterial susceptibility and resistance to these natural bioactive systems.

## 5. Conclusions

In conclusion, RDM and PDM showed measurable in vitro growth-inhibitory activity against selected clinically relevant Gram-negative bacteria, including isolates with important AMR mechanisms. The observed effect was isolate-dependent, supporting the concept of donkey milk as a functional bioactive dairy matrix with potential relevance in food microbiology, functional nutrition, and complementary antimicrobial research. However, donkey milk should not be regarded as a substitute for conventional antimicrobial therapy. Further studies are needed to identify the bioactive components responsible for the observed effects, clarify their mechanisms of action, and evaluate the biological relevance of these findings in more complex experimental models.

## Figures and Tables

**Table 1:** Clinical Gram-negative isolates included in the study.

Isolate Code	Species	Clinical Specimen	Detected Resistance Mechanism
A1	*E. coli*	Wound swab	CTX-M
A3	*K. oxytoca*	Wound swab	Not detected
A4	*K. pneumoniae*	Sputum	OXA-48
A6	*S. marcescens*	Tracheal aspirate	Not detected
C5	*K. pneumoniae*	Wound swab	KPC
C8	*E. coli*	Urine	Not detected
F4	*P. stuartii*	Wound swab	OXA-48
F5	*C. koseri*	Blood	NDM
F8	*E. coli*	Blood	NDM
F9	*P. mirabilis*	Blood	VIM

Note: CTX-M—type extended-spectrum β-lactamases; OXA-48, KPC, NDM, and VIM denote carbapenemase types. “Not detected” refers to the absence of ESBL or carbapenemase production as determined by the applied lateral flow assays.

**Table 2:** Clinical Gram-negative isolates and their antimicrobial resistance profiles.

Isolate Code	Species	Detected β-Lactamase	Main Resistance Features	MDR Phenotype
A1	*E. coli*	CTX-M	Cephalosporins, fluoroquinolones, aminoglycosides, trimethoprim/sulfamethoxazole	Yes
A3	*K. oxytoca*	Not detected	trimethoprim/sulfamethoxazole	No
A4	*K. pneumoniae*	OXA-48	Cephalosporins, carbapenems, fluoroquinolones, trimethoprim/sulfamethoxazole, aminoglycosides	Yes
A6	*S. marcescens*	Not detected	No acquired β-lactamase detected; susceptible to most tested agents	No
C5	*K. pneumoniae*	KPC	Broad β-lactam resistance, carbapenems, fluoroquinolones, aminoglycosides, trimethoprim/sulfamethoxazole	Yes
C8	*E. coli*	Not detected	Susceptible to most tested agents	No
F4	*P. stuartii*	OXA-48	Cephalosporins, carbapenems, fluoroquinolones, aminoglycosides, trimethoprim/sulfamethoxazole	Yes
F5	*C. koseri*	NDM	Broad β-lactam resistance, carbapenems, aminoglycosides, trimethoprim/sulfamethoxazole	Yes
F8	*E. coli*	NDM	Broad β-lactam resistance, carbapenems, fluoroquinolones, aminoglycosides, trimethoprim/sulfamethoxazole	Yes
F9	*P. mirabilis*	VIM	Broad β-lactam resistance, carbapenems, fluoroquinolones, aminoglycosides, trimethoprim/sulfamethoxazole	Yes

Note: CTX-M—type extended-spectrum β-lactamases. OXA-48, KPC, NDM, and VIM denote carbapenemase types. “Not detected” indicates that ESBL or carbapenemase production was not detected using the applied lateral flow assays. MDR phenotype was defined as non-susceptibility to at least one agent in three or more antimicrobial categories. Ceftazidime/avibactam results were interpreted descriptively; resistance is expected in NDM- and VIM-producing isolates because avibactam does not inhibit metallo-β-lactamases.

**Table 3:** Summary of two-way ANOVA results for the effects of milk type and incubation time on log-transformed bacterial count.

Isolate	Milk Type F (ω^2^)	Incubation Time F (ω^2^)	Milk Type × Incubation Time F (ω^2^)	Main Source of Variation Based on ω^2^
*Klebsiella pneumoniae* A4	9047 (0.337)	3240 (0.322)	1140 (0.340)	Comparable effects; interaction slightly highest
*Klebsiella oxytoca* A3	1140.6 (0.061)	6036.2 (0.862)	175.5 (0.075)	Incubation time
*Escherichia coli* C8	1412.3 (0.064)	7259.5 (0.875)	164.4 (0.059)	Incubation time
*Escherichia coli* A1	5970.4 (0.339)	2204.2 (0.334)	713.8 (0.324)	Comparable effects; no single dominant source
*Serratia marcescens* A6	993.1 (0.046)	7405.0 (0.911)	112.3 (0.041)	Incubation time
*Citrobacter koseri* F5	180.38 (0.016)	3995.48 (0.952)	40.75 (0.028)	Incubation time
*Escherichia coli* F8	5455.7 (0.235)	4775.0 (0.549)	621.5 (0.214)	Incubation time
*Proteus mirabilis* F9	1149.6 (0.113)	2887.0 (0.756)	163.1 (0.127)	Incubation time
*Providencia stuartii* F4	899.0 (0.073)	3713.3 (0.801)	192.6 (0.124)	Incubation time
*Klebsiella pneumoniae* C5	8126.8 (0.123)	19,589.4 (0.789)	722.2 (0.087)	Incubation time

Note: For all isolates, the effects of milk type, incubation time, and their interaction were statistically significant at *p* < 0.001. Note: The main source of variation was determined based on the largest ω^2^ value. When the ω^2^ values for milk type, incubation time, and their interaction were very similar, no single dominant effect was identified; instead, these effects were interpreted as having comparable contributions to the total variability.

## Data Availability

The data used to support the findings of this study are available in the present manuscript.

## Supplementary Materials

The following supporting information can be downloaded at https://www.mdpi.com/article/10.3390/ani16131996/s1: Table S1: Mean bacterial counts (log CFU/mL ± SD) of Gram-negative isolates during incubation in different matrices.

## Abbreviations

The following abbreviations are used in this manuscript:

AMR	Antimicrobial resistance
WHO	World Health Organization
MDR	Multidrug-resistant
ESBLs	Extended-spectrum β-lactamases
RDM	Raw donkey milk
PDM	Pasteurized donkey milk
CFU	Colony-forming units
LTLT	Low-temperature long-time
HTST	high-temperature short-time
NB	Nutrient broth
CM	Cow milk
VRBG	Violet Red Bile Glucose
SD	Standard deviation
ANOVA	Analysis of variance
HSD	Honestly significant difference
ω^2^	Omega-squared effect size coefficient
EUCAST	European Committee on Antimicrobial Susceptibility Testing
CTX-M	CTX-M-type extended-spectrum β-lactamase
OXA-48	Oxacillinase-48 carbapenemase
KPC	*Klebsiella pneumoniae* carbapenemase
NDM	New Delhi metallo-β-lactamase
VIM	Verona integron-encoded metallo-β-lactamase
