# Supplementary material for: Growth-Inhibitory Activity of Raw and Pasteurized Donkey Milk Against Clinically Relevant Gram-Negative Isolates with Different Antimicrobial Resistance Profiles
Source: Animals (Basel). 2026 Jun 29;16(13):1996. doi: 10.3390/ani16131996 (PMC13359499; doi:10.3390/ani16131996)
Supplement: Supplementary file 1 [file animals-16-01996-s001.zip › animals-4382173-supplementary.pdf]

Supplementary

Table 1. Mean bacterial counts (log CFU/mL ± SD) of Gram-negative isolates during incubation in different matrices.

Isolate	Time (h)	SDM	PDM	HB	KM
K. pneumoniae A4	0	2.230 ± 0.030	2.230 ± 0.010	2.203 ± 0.025	2.177 ± 0.025
K. pneumoniae A4	1	2.310 ± 0.020	2.243 ± 0.015	2.147 ± 0.035	2.147 ± 0.035
K. pneumoniae A4	2	2.377 ± 0.035	2.203 ± 0.025	2.543 ± 0.035	2.650 ± 0.050
K. pneumoniae A4	3	2.220 ± 0.040	2.450 ± 0.040	3.253 ± 0.050	2.077 ± 0.035
K. pneumoniae A4	4	2.340 ± 0.040	2.437 ± 0.055	3.557 ± 0.045	2.430 ± 0.050
K. pneumoniae A4	5	2.387 ± 0.035	2.553 ± 0.060	4.640 ± 0.060	4.130 ± 0.050
K. pneumoniae A4	6	2.360 ± 0.040	2.597 ± 0.065	5.410 ± 0.070	4.650 ± 0.050
K. pneumoniae A4	7	2.040 ± 0.040	2.587 ± 0.065	5.950 ± 0.050	5.110 ± 0.050
K. pneumoniae A4	8	1.697 ± 0.045	2.597 ± 0.085	6.253 ± 0.050	5.520 ± 0.040
K. oxitoca A3	0	1.953 ± 0.025	1.990 ± 0.010	1.977 ± 0.025	2.020 ± 0.000
K. oxitoca A3	1	2.007 ± 0.031	1.963 ± 0.071	2.057 ± 0.055	2.000 ± 0.020
K. oxitoca A3	2	1.930 ± 0.030	2.237 ± 0.060	2.420 ± 0.085	2.393 ± 0.081
K. oxitoca A3	3	2.657 ± 0.060	2.660 ± 0.137	2.797 ± 0.100	2.620 ± 0.000
K. oxitoca A3	4	3.580 ± 0.040	3.677 ± 0.025	3.507 ± 0.055	3.750 ± 0.082
K. oxitoca A3	5	3.963 ± 0.035	3.727 ± 0.080	4.643 ± 0.071	4.610 ± 0.000
K. oxitoca A3	6	4.157 ± 0.045	4.067 ± 0.076	5.343 ± 0.065	5.300 ± 0.100
K. oxitoca A3	7	4.307 ± 0.050	4.230 ± 0.098	6.117 ± 0.060	5.727 ± 0.031
K. oxitoca A3	8	4.403 ± 0.045	4.327 ± 0.031	6.663 ± 0.045	6.123 ± 0.051
E. coli C8	0	2.000 ± 0.000	2.060 ± 0.000	2.000 ± 0.020	2.007 ± 0.031
E. coli C8	1	1.990 ± 0.010	2.070 ± 0.036	2.000 ± 0.020	1.987 ± 0.035
E. coli C8	2	2.000 ± 0.000	1.833 ± 0.075	2.000 ± 0.000	2.313 ± 0.061
E. coli C8	3	2.053 ± 0.025	1.990 ± 0.000	2.010 ± 0.066	2.483 ± 0.075
E. coli C8	4	2.563 ± 0.045	2.913 ± 0.085	3.013 ± 0.091	3.483 ± 0.075
E. coli C8	5	3.213 ± 0.042	3.810 ± 0.090	3.960 ± 0.115	4.490 ± 0.085
E. coli C8	6	3.677 ± 0.045	4.483 ± 0.105	5.027 ± 0.112	5.487 ± 0.090
E. coli C8	7	4.047 ± 0.050	4.950 ± 0.000	6.300 ± 0.000	6.110 ± 0.000
E. coli C8	8	4.300 ± 0.040	5.270 ± 0.095	6.480 ± 0.080	6.603 ± 0.065
E. coli A1	0	2.223 ± 0.021	2.290 ± 0.066	2.210 ± 0.066	2.260 ± 0.060
E. coli A1	1	2.307 ± 0.015	2.240 ± 0.010	2.147 ± 0.035	2.283 ± 0.055
E. coli A1	2	2.270 ± 0.010	2.203 ± 0.095	2.547 ± 0.070	2.650 ± 0.070
E. coli A1	3	2.217 ± 0.015	2.440 ± 0.026	3.260 ± 0.065	2.087 ± 0.090
E. coli A1	4	2.340 ± 0.020	2.447 ± 0.070	3.563 ± 0.055	2.440 ± 0.066
E. coli A1	5	2.390 ± 0.020	2.553 ± 0.070	4.647 ± 0.070	4.137 ± 0.060
E. coli A1	6	2.480 ± 0.030	2.603 ± 0.065	5.420 ± 0.085	4.657 ± 0.060
E. coli A1	7	2.040 ± 0.020	2.600 ± 0.000	5.900 ± 0.000	5.117 ± 0.060
E. coli A1	8	1.903 ± 0.025	2.620 ± 0.072	6.217 ± 0.076	5.527 ± 0.050
S. marcescens A6	0	1.880 ± 0.030	1.900 ± 0.040	1.907 ± 0.050	1.997 ± 0.045
S. marcescens A6	1	1.907 ± 0.031	1.950 ± 0.040	1.873 ± 0.021	1.977 ± 0.045
S. marcescens A6	2	1.960 ± 0.056	1.940 ± 0.056	2.057 ± 0.086	2.077 ± 0.075
S. marcescens A6	3	2.050 ± 0.075	2.093 ± 0.081	2.303 ± 0.105	2.447 ± 0.065
S. marcescens A6	4	2.693 ± 0.065	3.003 ± 0.075	3.553 ± 0.070	3.387 ± 0.070

S. marcescens A6	5	3.607 ± 0.070	4.303 ± 0.075	4.453 ± 0.055	4.313 ± 0.061
S. marcescens A6	6	3.987 ± 0.080	4.683 ± 0.065	5.313 ± 0.091	5.007 ± 0.031
S. marcescens A6	7	4.113 ± 0.035	5.130 ± 0.050	5.950 ± 0.000	5.110 ± 0.000
S. marcescens A6	8	4.207 ± 0.031	5.437 ± 0.040	6.607 ± 0.050	5.810 ± 0.050
Citrobacter koserii F5	0	2.000 ± 0.020	1.950 ± 0.050	1.910 ± 0.066	1.953 ± 0.025
Citrobacter koserii F5	1	1.963 ± 0.071	1.953 ± 0.025	1.853 ± 0.075	1.950 ± 0.010
Citrobacter koserii F5	2	2.080 ± 0.020	2.010 ± 0.066	1.963 ± 0.071	2.010 ± 0.066
Citrobacter koserii F5	3	2.283 ± 0.117	2.330 ± 0.075	2.440 ± 0.066	2.507 ± 0.055
Citrobacter koserii F5	4	2.853 ± 0.045	3.087 ± 0.090	4.003 ± 0.105	3.183 ± 0.075
Citrobacter koserii F5	5	3.320 ± 0.151	3.947 ± 0.095	3.893 ± 0.110	3.847 ± 0.065
Citrobacter koserii F5	6	3.473 ± 0.060	4.303 ± 0.075	4.493 ± 0.101	4.677 ± 0.186
Citrobacter koserii F5	7	4.697 ± 0.095	4.600 ± 0.020	5.010 ± 0.066	5.390 ± 0.046
Citrobacter koserii F5	8	5.340 ± 0.020	5.300 ± 0.040	5.900 ± 0.050	6.307 ± 0.050
E. coli F8	0	1.480 ± 0.030	1.953 ± 0.025	2.000 ± 0.000	1.953 ± 0.025
E. coli F8	1	1.920 ± 0.151	2.043 ± 0.065	2.077 ± 0.035	1.900 ± 0.000
E. coli F8	2	2.650 ± 0.020	2.600 ± 0.020	2.777 ± 0.035	1.997 ± 0.045
E. coli F8	3	2.643 ± 0.071	2.627 ± 0.070	2.977 ± 0.035	2.727 ± 0.050
E. coli F8	4	2.477 ± 0.015	2.723 ± 0.025	4.113 ± 0.035	3.340 ± 0.040
E. coli F8	5	2.717 ± 0.126	2.793 ± 0.101	4.533 ± 0.025	3.910 ± 0.030
E. coli F8	6	2.020 ± 0.082	3.123 ± 0.051	5.447 ± 0.035	4.493 ± 0.045
E. coli F8	7	2.963 ± 0.071	3.313 ± 0.061	6.557 ± 0.025	4.477 ± 0.015
E. coli F8	8	3.300 ± 0.010	3.603 ± 0.006	7.040 ± 0.020	6.340 ± 0.040
Proteus mirabilis F9	0	2.000 ± 0.010	2.077 ± 0.035	2.047 ± 0.031	1.950 ± 0.010
Proteus mirabilis F9	1	2.003 ± 0.015	2.000 ± 0.020	2.007 ± 0.031	2.320 ± 0.181
Proteus mirabilis F9	2	2.003 ± 0.025	1.907 ± 0.031	2.000 ± 0.010	2.300 ± 0.010
Proteus mirabilis F9	3	1.850 ± 0.040	2.007 ± 0.031	2.003 ± 0.055	2.473 ± 0.060
Proteus mirabilis F9	4	2.007 ± 0.031	1.960 ± 0.036	2.307 ± 0.050	2.903 ± 0.055
Proteus mirabilis F9	5	2.903 ± 0.055	2.960 ± 0.036	3.307 ± 0.050	3.300 ± 0.010
Proteus mirabilis F9	6	2.950 ± 0.040	2.950 ± 0.040	4.003 ± 0.055	3.600 ± 0.040
Proteus mirabilis F9	7	3.007 ± 0.050	2.997 ± 0.045	4.657 ± 0.060	3.950 ± 0.010
Proteus mirabilis F9	8	3.280 ± 0.020	3.347 ± 0.117	5.307 ± 0.050	4.247 ± 0.031
Providencia stuartii F4	0	1.953 ± 0.025	1.953 ± 0.025	1.903 ± 0.015	1.903 ± 0.015
Providencia stuartii F4	1	2.000 ± 0.030	1.847 ± 0.035	1.910 ± 0.066	1.903 ± 0.045
Providencia stuartii F4	2	1.950 ± 0.030	2.000 ± 0.020	2.000 ± 0.010	1.897 ± 0.015
Providencia stuartii F4	3	1.997 ± 0.035	1.953 ± 0.025	1.877 ± 0.112	2.070 ± 0.066
Providencia stuartii F4	4	1.997 ± 0.025	1.847 ± 0.035	2.077 ± 0.035	1.853 ± 0.075
Providencia stuartii F4	5	2.297 ± 0.035	2.300 ± 0.020	2.870 ± 0.072	2.713 ± 0.071
Providencia stuartii F4	6	2.997 ± 0.035	2.700 ± 0.020	3.700 ± 0.020	3.307 ± 0.050
Providencia stuartii F4	7	3.010 ± 0.036	2.980 ± 0.072	4.853 ± 0.075	3.600 ± 0.060
Providencia stuartii F4	8	3.300 ± 0.030	3.300 ± 0.020	4.950 ± 0.010	3.907 ± 0.031
K. pneumoniae C5	0	1.847 ± 0.035	1.953 ± 0.015	1.847 ± 0.015	2.000 ± 0.010
K. pneumoniae C5	1	1.900 ± 0.000	1.850 ± 0.050	1.900 ± 0.020	2.043 ± 0.015
K. pneumoniae C5	2	2.230 ± 0.050	2.480 ± 0.060	3.600 ± 0.030	2.083 ± 0.025
K. pneumoniae C5	3	2.783 ± 0.045	3.080 ± 0.100	4.397 ± 0.035	2.970 ± 0.030
K. pneumoniae C5	4	3.277 ± 0.045	3.603 ± 0.045	4.917 ± 0.035	3.783 ± 0.025
K. pneumoniae C5	5	3.453 ± 0.055	3.807 ± 0.045	5.650 ± 0.030	4.823 ± 0.035
K. pneumoniae C5	6	3.807 ± 0.045	4.600 ± 0.040	5.650 ± 0.030	5.560 ± 0.000

K. pneumoniae C5	7	3.940 ± 0.030	5.603 ± 0.045	6.447 ± 0.035	6.480 ± 0.030
K. pneumoniae C5	8	4.077±0.035	6.480±0.050	7.377±0.035	7.267±0.021
